# The Impact of Frequency Scale on the Response Sensitivity and Reliability of Cortical Neurons to 1/f^β^ Input Signals

**DOI:** 10.3389/fncel.2019.00311

**Published:** 2019-07-11

**Authors:** Guojie Qu, Boqiang Fan, Xin Fu, Yuguo Yu

**Affiliations:** State Key Laboratory of Medical Neurobiology, School of Life Science, Human Phenome Institute, Institute of Brain Science, Institute of Science and Technology for Brain-Inspired Intelligence, Fudan University, Shanghai, China

**Keywords:** 1/f^β^ noise, cortical neurons, patch clamp recording, long-term correlation, Hodgkin-Huxley model, response reliability

## Abstract

What type of principle features intrinsic inside of the fluctuated input signals could drive neurons with the maximal excitations is one of the crucial neural coding issues. In this article, we examined both experimentally and theoretically the cortical neuronal responsivity (including firing rate and spike timing reliability) to input signals with different intrinsic correlational statistics (e.g., white-type noise, showed 1/f^0^ power spectrum, pink noise 1/f, and brown noises 1/f^2^) and different frequency ranges. Our results revealed that the response sensitivity and reliability of cortical neurons is much higher in response to 1/f noise stimuli with long-term correlations than 1/f^0^ with short-term correlations for a broad frequency range, and also higher than 1/f^2^ for all frequency ranges. In addition, we found that neuronal sensitivity diverges to opposite directions for 1/f noise comparing with 1/f^0^ white noise as a function of cutoff frequency of input signal. As the cutoff frequency is progressively increased from 50 to 1,000 Hz, the neuronal responsiveness increased gradually for 1/f noise, while decreased exponentially for white noise. Computational simulations of a general cortical model revealed that, neuronal sensitivity and reliability to input signal statistics was majorly dominated by fast sodium inactivation, potassium activation, and membrane time constants.

## Introduction

For a signal, the inherent frequency structure shown in the Fourier frequency domain characterizes its second-order statistics. The power spectrum of various natural signals typically exhibits the power law 1/f^β^ in the frequency domain, with β close to one (Voss and Clarke, 1978; Gilden et al., 1995; Musha and Yamamoto, 1997; De Coensel et al., 2003). Moreover, this 1/f property within a specific frequency range is widely observed in neural activities at all levels, as evidenced in recordings of the membrane potential and current (Diba, 2004; Jacobson et al., 2005; Bédard et al., 2006; Yaron-Jakoubovitch, 2008; El Boustani et al., 2009), EEG (Novikov et al., 1997; Bhattacharya and Petsche, 2001; Bédard et al., 2006; Dehghani et al., 2010; Voytek et al., 2015), MEG (Novikov et al., 1997; Dehghani et al., 2010), LFPs (Bédard and Destexhe, 2009; Bedard et al., 2017; Maex, 2018), and fMRI signals (Bullmore et al., 2001; He, 2011; Ciuciu et al., 2014). In practice, the white noise (the power law 1/f^0^) with generally low cutoff frequencies is widely used to detect neuronal input-and-output functions (Sakai, 1992; Fairhall et al., 2001; Cook et al., 2007; Vilela and Lindner, 2009). Besides, 1/f^2^ noise is also observed in neural field potentials (Freeman and Zhai, 2009; Miller et al., 2009; Milstein et al., 2009; He et al., 2010; Halnes et al., 2016) and membrane currents under special conditions (Diba, 2004).

Mammalian sensory neural systems exhibit better responses to naturalistic signals rather than white-type noise signals in a specific frequency domain (Aertsen and Johannesma, 1981; Baddeley et al., 1997; de Ruyter van Steveninck et al., 1997; Yu et al., 2005; Garcia-Lazaro et al., 2006, 2011). It has been speculated that the 1/f property might be the key in shaping the neuronal function preference to the naturalistic input (Yu et al., 2005; Garcia-Lazaro et al., 2006, 2011); this preference may extend to the atomic level of neural organization, namely the single neuronal input-output function (Gal and Marom, 2013).

In addition, white and colored noises with various cutoff frequencies enhance the detection of weak signals by neuronal systems via stochastic resonance (SR) (Nozaki and Yamamoto, 1998,?; Nozaki et al., 1999b; Hutcheon and Yarom, 2000; Jia et al., 2001; Ruszczynski et al., 2001; Chizhov and Graham, 2008; Mino and Durand, 2008; Gutkin et al., 2009; Higgs and Spain, 2009; Sekine et al., 2009; Guo and Li, 2011; Sobie et al., 2011; Duan et al., 2014; Zhao et al., 2017). However, previous studies have regarded the signals with 1/f^β^ statistics as mere background noise. In particular, although the signal frequency range has been confirmed to significantly affect neuronal excitability (Nozaki et al., 1999b; Higgs and Spain, 2009), the exact role of the frequency range in neuronal responsiveness to the 1/f^β^ statistic remains unclear. As neurons generally encode information according to the rate and/or the precise timing of spikes (Nowak et al., 1997; Reinagel and Reid, 2000; Fellous et al., 2001; Brette and Guigon, 2003; Avissar et al., 2007; Freund and Cerquera, 2012), we sought to examine the neuronal firing rate and spike-timing reliability to input statistics. In this study, we focus on the effects of the signal frequency range to identify the neuronal responsiveness to 1/f^β^ (β = 0, 1, and 2) noises with respect to the firing rate and spike-timing reliability. Specifically, we reveal the mechanism underlying neuronal responsivity to 1/f^β^ and the frequency range using theoretical experiments, as this mechanism has not been clearly determined in previous model-based studies (Nozaki and Yamamoto, 1998; Nozaki et al., 1999b; Brunel and Latham, 2003; Mino and Durand, 2008; Sekine et al., 2009; Sobie et al., 2011; Ostojic et al., 2015; Schwalger et al., 2015; Zhao et al., 2017). We conducted *in vitro* whole-cell patch clamp recording experiments on mouse cortical pyramidal neurons to examine the neuronal firing rate and reliability to 1/f, 1/f^0^, and 1/f^2^ noises with various cutoff frequencies (F_cut_). We have also carried out a set of computational simulations of a general Hodgkin-Huxley neuronal model (Yu et al., 2012) to reproduce our experimental observations, and revealed the critical factors underlying the neuronal responsiveness to second-order statistics at the cellular level.

## Methods

### Signal Production

**Figure 1:**
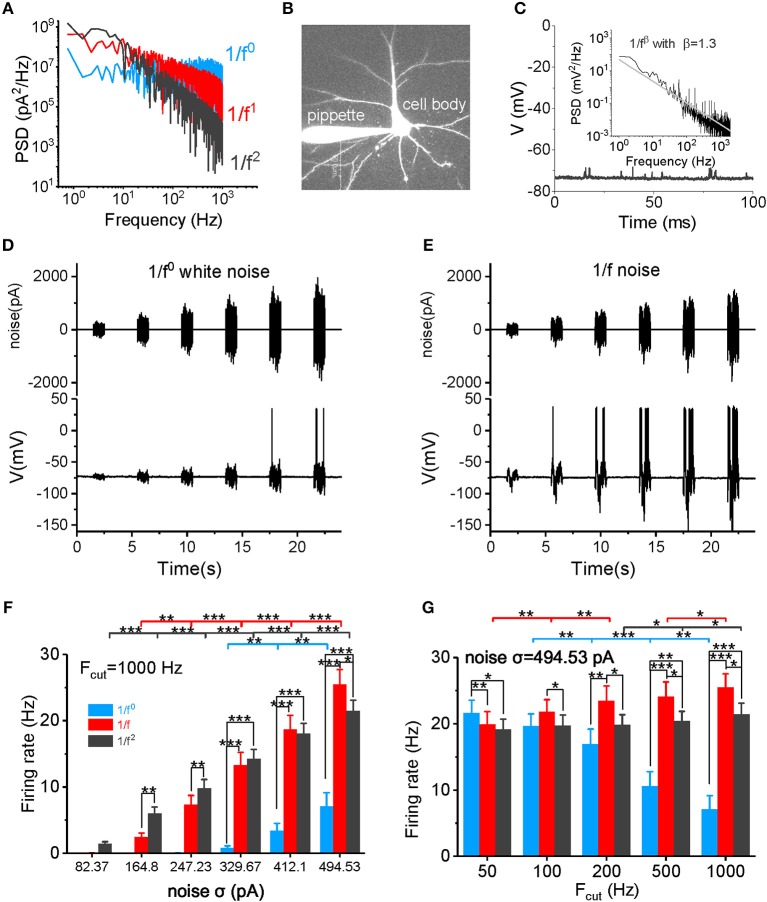
Neuronal firing rate in response to different types of input signal. **(A)** The PSD plots for white noise (blue), 1/f noise (red), and 1/f^2^ noise (black) are shown. Noises with a 1,000 Hz cutoff frequency are shown here. **(B)** *In vitro* patch clamp recording of a pyramidal cell. The scale bar indicates 50 μm. **(C)** Plot of the recorded membrane potentials in the resting state vs. time. Inset: The power spectrum density (PSD) plot of the resting potentials shows a 1/f property in a log-log plot. **(D,E)** Representative trace of input 1/f^0^ white noise **(D)** and 1/f noise **(E)** with different intensities over time. The signal produced at each intensity lasted for 1 s, followed by a 3 s no-stimulus interval. The bottom panel shows the membrane potential and action potential produced by a recorded pyramidal cell in response to the input signal shown in the top panel. **(F)** Neuronal responsive firing rate for white noise (blue), 1/f (red), and 1/f^2^ noise (black) with various intensities (82.37, 164.80, 247.23, 329.67, 412.10, and 494.53 pA) and a cutoff frequency of 1000 Hz. F_cut_, cutoff frequency. **(G)** Neuronal responsive firing rate for white noise (blue), 1/f (red), and 1/f^2^ noise (black) at various cutoff frequencies (50, 100, 200, 500, and 1,000 Hz) with a intensity of 494.53 pA.

Input noise stimuli of each type of 1/f^β^–white noise (1/f^0^), pink noise (1/f), and brown noise (1/f^2^) were first generated digitally by computer programming. The noise stimuli were then filtered by different low-pass filter with aimed cutoff frequency range. Each signal intensity [represented by the standard deviation (SD)] was set to the defined value. All the above were done in Matlab R2017a software (Mathworks, USA), and then the signal was loaded to Micro 1401 (CED, UK), where the signals were converted from digital to analog. In the experimental study, a unique set of noise stimuli with designed cutoff frequencies and noise intensities were used as the input signals to recorded neurons. The three signal types have different slopes in PSDs (the white noise has a slope of 0 and 1/f noise has a slope of −1, while 1/f^2^ noise has a slope of −2), as shown in Figure 1A. The 1/f and 1/f^2^ noises have more power in the low frequency components and less power in the high frequency components, while white noise has equal energy at each frequency interval. All three types of signals have equal total power within the examined frequency range.

### Brain Slice Preparation

The experimental procedures involving animal experiments in this study were approved by Animal Ethics Committee of Fudan University School of Life Science. Using 0.7% chloral hydrate, 14–28 day-old mice were anesthetized and brain slices prepared by a protective slicing and recovery method reported previously (Ting et al., 2014). Briefly, anesthetized mice were perfused intracardially with ice-cold oxygenated (95% O_2_, 5% CO_2_) NMDG-based cutting solution containing (in mM) 93 mM NMDG, 2.5 mM KCl, 1.2 mM NaH_2_PO_4_, 30 mM NaHCO_3_, 20 mM HEPES, 25 mM glucose, 5 mM sodium ascorbate, 2 mM thiourea, 3 mM sodium pyruvate, 10 mM MgSO_4_, 2 mM CaCl_2_, and 12 mM NAC (pH 7.3–7.4, 300–305 mOsm). Brains were carefully removed from the skull and cut coronally at a thickness of 300 μm with a vibratome VT1000S (Leica, Germany) in chilled oxygenated (95% O_2_, 5% CO_2_) NMDG-based cutting solution. Slices were initially recovered in NMDG-based cutting solution at 32°C for 10 min. Slices were then incubated in oxygenated (95% O_2_, 5% CO_2_) HEPES-modified solution containing (in mM) 94 mM NaCl, 2.5 mM KCl, 1.2 mM NaH_2_PO_4_, 30 mM NaHCO_3_, 20 mM HEPES, 25 mM glucose, 5 mM sodium ascorbate, 2 mM thiourea, 3 mM sodium pyruvate, 2 mM MgSO_4_, 2 mM CaCl_2_, and 6 mM NAC (pH 7.3–7.4, 300–305 mOsm) at room temperature for 30 min. Finally, slices were incubated in oxygenated (95% O_2_, 5% CO_2_) ACSF at room temperature for at least 1 h before recording. The ACSF contained 126 mM NaCl, 2.5 mM KCl, 2 mM MgSO_4_, 2 mM CaCl_2_, 26 mM NaHCO_3_, 1.25 mM NaH_2_PO_4_, and 12.5 mM dextrose (pH 7.3–7.4, 300–305 mOsm).

### Electrophysiological Recordings

Whole-cell slice recordings were performed on the cell body of layer 5 pyramidal neurons in the prefrontal cortex (Figure 1B). In total, 12 neurons in 12 slices of six mice were examined. Oxygenated (95% O_2_, 5% CO_2_) ASCF was used as the recording solution. Recordings were conducted on an upright infrared-differential interference contrast (IR-DIC) microscope (Zeiss Axioskop 2 FS plus) with a recording solution temperature of 36 °C. Cortical slices were suspended on a net to allow an oxygenated solution to flow over both the upper and lower surfaces at a rate of 3–4 ml/min. The membrane potential in the whole-cell recordings was corrected for Donnan liquid junction potentials of 15 mV. The temperature was regulated by a Warner Instruments Corporation two channel temperature regulator (Model TC344B).

Whole-cell recordings from the soma were achieved with the help of a Multiclamp 700B amplifier (Axon Instruments, Union City, CA) and Micro 1401 converter. Pipettes had an impedance of 5–6 MΩ and were filled with an intracellular solution that contained 140 mM K-gluconate, 3 mM KCl, 2 mM MgCl_2_, 2 mM Na_2_ATP, 10 mM HEPES, and 0.2 mM EGTA, and the pH was adjusted to 7.3 with KOH–(270 mOsm). The sample rate of the membrane potential data was 25 kHz for both experiments and computational simulations. Those recorded neurons with input resistance within 100–200 MΩ were saved for analysis in this paper while others were discarded so that the studied neurons have the similar biophysical membrane properties.

### Stimulation Mode

To experimentally examine the responsive firing rate to the input signal, the current signal with each intensity (σ = 82.37, 164.80, 247.23, 329.67, 412.10, and 494.53 pA) and each cutoff frequency (F_cut_ = 50, 100, 200, 500, and 1,000 Hz) was injected into neurons three times (Figures 1D,E). The signals (σ = 494.53 pA; F_cut_ = 50, 200, 500, and 1,000 Hz) in firing rate detection were also used for spike reliability examination, in which 50 repetitions of signals were injected into neurons. Every neuron in our study received all types of input signal, with its firing rate and reliability for each signal cutoff frequency examined. In the model simulation, the neuron received input signals (σ = 1.5, 3, 4.5, 6, 7.5, 9 μA/cm^2^; Fcut = 50, 200, 500, and 1,000 Hz) thrice for firing rate detection. The signal (σ = 9 pA; F_cut_ = 50, 200, 500, and 1,000 Hz) was also injected 50 times for the spike reliability examination. In each test, to simulate the background noise from external or intrinsic fluctuations of the neuron itself, a 1/f noise with 500 Hz cutoff frequency and an intensity of 0.2169 μA/cm^2^ was added to the input signal. The duration of stimuli signal of each cutoff frequency and intensity was 1 s.

### Firing Rate and Spike Time Reliability

For each neuron under an input signal of each cutoff frequency and intensity, the spike number within the 1s stimuli duration was the firing rate. We averaged the firing rates in the three repetitions to get each neuronal firing rate value. For spike time reliability, 50 repetitions of a 1 s stimuli were injected into neurons, and cross covariance was calculated between all pair-wise combinations of trials (within stimulus size) within each neuron, on binary spike timing trains (with “1” representing an action potential with a 2 ms time bin and “0” representing a non-spiking neuron). The black-covariance function estimates the mean-removed cross-correlation between the two sequences of random processes, thus avoiding the contribution of the mean firing rate to the spike timing reliability. The resulting cross-covariance values at zero-lag (normalized by the average autocovariance function) for each neuron were used to quantify the spike reliability for each input signal statistic (Haider et al., 2010).

### Hodgkin-Huxley-Style Cortical Neuronal Model

Three major ionic voltage-dependent currents were used in our cortical model: fast Na^+^, I_Na_, fast K^+^, I_K_, and a leak current, I_L_. The equations describing the voltage and time dependence of the Na^+^ and K^+^ conductance have been reported in previous publications (McCormick and Huguenard, 1992) and the channel kinetics were modified based on models of cortical neurons (Mainen et al., 1995; Mainen and Sejnowski, 1996; Yu et al., 2008) and experimental studies (Huguenard et al., 1989; Colbert and Pan, 2002; Yu et al., 2008; Schmidt-Hieber and Bischofberger, 2010). The following equations describe the cortical axon single compartment model:

$$\begin{array}{l} C\frac{\text{dV}}{\text{dt}}=I_{stim}-g_{Na}^{max}\cdot m^{3}\cdot h\cdot \left(V-V_{Na}\right)\\ \;-g_{K}^{max}\cdot n\cdot \left(V-V_{K}\right)-g_{L}\cdot \left(V-V_{L}\right),\\ \tau m\frac{dm}{dt}=-m+m_{\infty },\;\tau m=\frac{1}{\alpha_{m}+\beta_{m}},\;m_{\infty }=\frac{\alpha_{m}}{\alpha_{m}+\beta_{m}}\\ \tau h\frac{dh}{dt}=-h+h_{\infty },\;\tau h=\frac{1}{\alpha_{h}+\beta_{h}},\;h_{\infty }=\frac{1}{1+e^{\left(V+60\right)/6.2}}\\ \tau n\frac{dn}{dt}=-n+n_{\infty },\;\tau_{n}=\frac{1}{\alpha_{n}+\beta_{n}},\;n_{\infty }=\frac{\alpha_{n}}{\alpha_{n}+\beta_{n}}\\ \alpha_{m}\left(V\right)=\emptyset \cdot \frac{0.182\cdot \left(V+30\right)}{1-\;e^{-\left(V+30\right)/8}}\\ \beta_{m}\left(V\right)=-\emptyset \cdot \frac{0.124\cdot \left(V+30\right)}{1-\;e^{\left(V+30\right)/8}}\\ \alpha_{h}\left(V\right)=\emptyset \cdot \frac{0.028\cdot \left(V+45\right)}{1-\;e^{-\left(V+45\right)/6}}\\ \beta_{h}\left(V\right)=-\emptyset \cdot \frac{0.0091\cdot \left(V+70\right)}{1-\;e^{\left(V+70\right)/6}}\\ \alpha_{n}\left(V\right)=\emptyset \cdot \frac{0.01\cdot \left(V-30\right)}{1-\;e^{-\left(V-30\right)/9}}\\ \beta_{n}\left(V\right)=-\emptyset \cdot \frac{0.0005\cdot \left(V-30\right)}{1-\;e^{\left(V-30\right)/9}}\\ \;\emptyset ={Q_{10}}^{\left(T-23\right)/10} \end{array}$$

where the Q_10_ effect is described by Φ on regulating the temperature dependence of the biochemical reaction rate with Q_10_ = 2.3 (Frankenhaeuser and Moore, 1963; Matteson and Armstrong, 1982). The relationships between temperature and I_Na_ and I_K_ activation and inactivation are not monotonic and vary in different species (Fohlmeister et al., 2010). The reverse potentials for Na^+^ and K^+^ currents were adjusted for change in temperature according to the Nernst equation (not shown). Similar results were obtained with a variety of values for Q_10_. For example, the use of a Q_10_ of 3 yielded similar results for spike efficiency and changes in spike rate with temperature. In our cortical model, Na^+^ kinetics were determined based on recent experimental observations (Kole et al., 2008). The parameters used were: membrane capacitance = 0.75 μF/cm^2^, g_Na_ = 1950 pS/μm^2^, density of g_K_ = 40 pS/μm^2^, and g_leak_ = 0.25 pS/μm^2^, based on recent experimental results (Mainen and Sejnowski, 1995; Colbert and Pan, 2002; Kole et al., 2008; Hu et al., 2009; Fleidervish et al., 2010; Schmidt-Hieber and Bischofberger, 2010). The reversal potentials were V_L_ = −70 mV, V_Na_ = 60 mV, and V_K_ = −90 mV for leak, sodium, and potassium channels, respectively.

### Statistical Analysis

To detect the differential response under various signal intensities, cutoff frequencies, or signal types, we performed paired *t*-test and Wilcoxon rank sum test. First, Kolmogorov-Smirnov goodness-of-fit hypothesis test was done on the paired difference between the two compared data groups. Depending on whether the normality assumption held for the data, we used a paired *t*-test or the Wilcoxon test for statistical significant comparison. A *p* < 0.05 was considered statistically significant. The data presented in the figures are reported as the mean ± standard error, and the significant level are labeled by ^*^p < 0.05, ^**^p < 0.01, and ^***^p < 0.001, respectively.

## Results

### Neuronal Responsiveness to Input Stimuli

In the absence of external stimuli, neuronal resting membrane potentials (~-75 to −70 mV) showed fluctuating synaptic potentials that were received from somatic and dendritic synapses (Figure 1C). A power spectrum density (PSD) analysis of resting potentials displayed a typical 1/f property in a log-log plot (see the inset in Figure 1C), suggesting that the membrane potentials observed *in vitro* in neurons with synaptic inputs also exhibit a similar statistical property as neurons *in vivo*.

First, we examined the neuronal firing rate in response to the input signal (Figures 1D,E). As shown in Figure 1F and Supplementary Figure S1, the neuronal firing rate increases non-linearly as the noise intensity increases within a firing rate of 0–30 Hz. When the noise cutoff frequency is within 50 Hz, neurons are sensitive to white noise, firing with a high frequency at high noise intensities (Figure 1G and Supplementary Figure S1A). However, as the cutoff frequency increases, neurons lose their responsiveness to the white noise (Figure 1G and Supplementary Figure S1) and produce a low firing rate, even at a very high noise intensity levels and when the cutoff frequency is 1,000 Hz (Figures 1F,G). The neuronal firing rate increases minimally at a cutoff frequency of 1/f^2^ noise (Figure 1G). In contrast, the firing rate increases gradually with the cutoff frequency for 1/f noise (Figure 1G). When the cutoff frequency is >200 Hz, firing rates for 1/f and 1/f^2^ noise are higher than for the white noise (Figures 1F,G, and Supplementary Figures S1C,D). At low intensities, 1/f noise evokes lower firing rates than the 1/f^2^ noise; but at high intensities, 1/f noise evokes higher firing rates than the 1/f^2^ noise. The switch in firing rates emerges under various cutoff frequencies (Figure 1F and Supplementary Figure S1). Notably, at σ = 494.53 pA and a 1,000 Hz cutoff frequency, 1/f noise evokes the highest firing rate (25.47 ± 2.24 Hz) in all our recordings. Overall, 1/f noise evokes relatively high firing rates under all conditions, particularly at high input intensities, and high cutoff frequencies.

**Figure 2:**
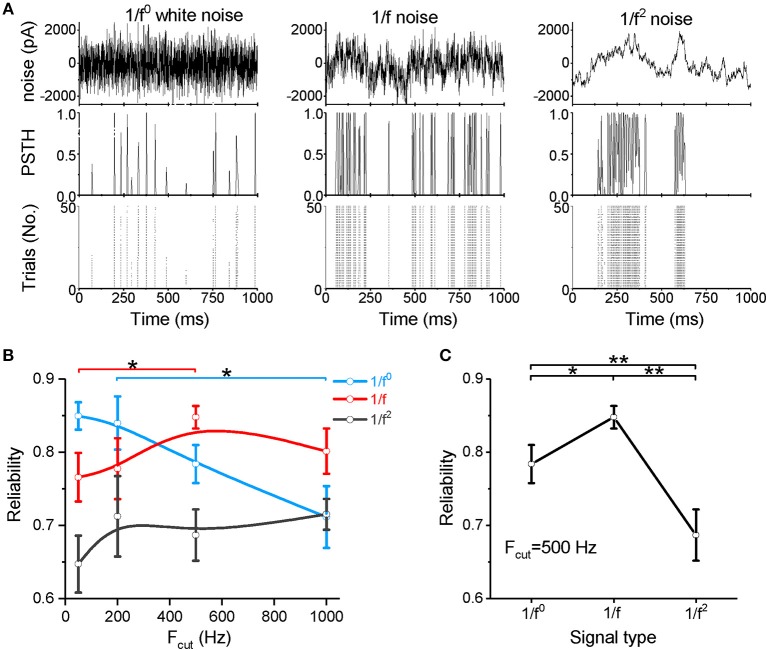
Neuronal spike reliability to different types of input signal. **(A)** Raster plot of the responses from 50 trials (bottom panel) and PSTH (middle panel) for the repeated input stimulus (top panel) reflecting the reliability of cortical neuron firing patterns evoked by white noise (left panel), 1/f noise (middle panel), and 1/f^2^ noise (right panel). **(B)** Plot of reliability vs. cutoff frequencies for the three signal types. F_cut_, cutoff frequency. **(C)** Plot of reliability vs. β for the three types of 1/f^β^ input signals with a cutoff frequency of 500 Hz.

We next examined spike reliability (Figure 2A) at four cutoff frequencies (F_cut_ = 50, 200, 500, and 1,000 Hz). The neuronal responses are shown in the post-stimulus time histogram (PSTH) and raster plot (Figure 2A). Figures 2B,C show the spike-timing reliability, which quantifies the extent of repeatability of spike timing in response to the input signal (Mainen and Sejnowski, 1995; Haider et al., 2010) (calculated from the representative data shown in Figure 1A). As the cutoff frequency increases to above 200 Hz, the reliability of the response to white noise decreases significantly (Figure 2B). For both the 1/f and 1/f^2^ noises, the reliability increases as the cutoff frequency increases (Figure 2B). When the cutoff frequency is 50 Hz, the reliability for the 1/f noise is greater than the 1/f^2^ noise and less than that for the white noise (Figure 2B and Supplementary Figure S2A). When the cutoff frequency increases to 200 Hz, the reliability for white noise remains greater than for the 1/f and 1/f^2^ noises (Figure 2B and Supplementary Figure S2B). Interestingly, at 500 and 1,000 Hz, the 1/f noise evokes the greatest reliability (Figures 2B,C, and Supplementary Figure S2C), suggesting that neurons respond with relatively high reliability to the 1/f property in signals, particularly when the input signal has a high cutoff frequency.

Our data demonstrate that neurons respond to white noise with low sensitivity and reliability, except when the noise is composed of low frequency components only. When the noise intensity is weak, 1/f^2^ noise more readily evokes neuronal firing, but high firing rate is difficult to evoke. For the 1/f noise, neurons respond with relatively high sensitivity and high reliability under all conditions. In particular, at high cutoff frequencies or high input intensities, the 1/f noise evokes the highest firing rate and the most reliable neuronal response among all signal types tested. These findings indicate a clear neuronal preference for responses to 1/f noise with high cutoff frequencies.

### Computer Simulations of Hodgkin-Huxley Model

A cortical Hodgkin-Huxley-type neuronal model was constructed to study the key factors and dynamic mechanisms underlying neuronal responsiveness to signals with different statistics. We only considered the fast sodium and potassium channels in the model and ignored other subtypes of sodium/potassium and calcium channels to identify the common mechanism underlying the neuronal preference for 1/f statistics.

**Figure 3:**
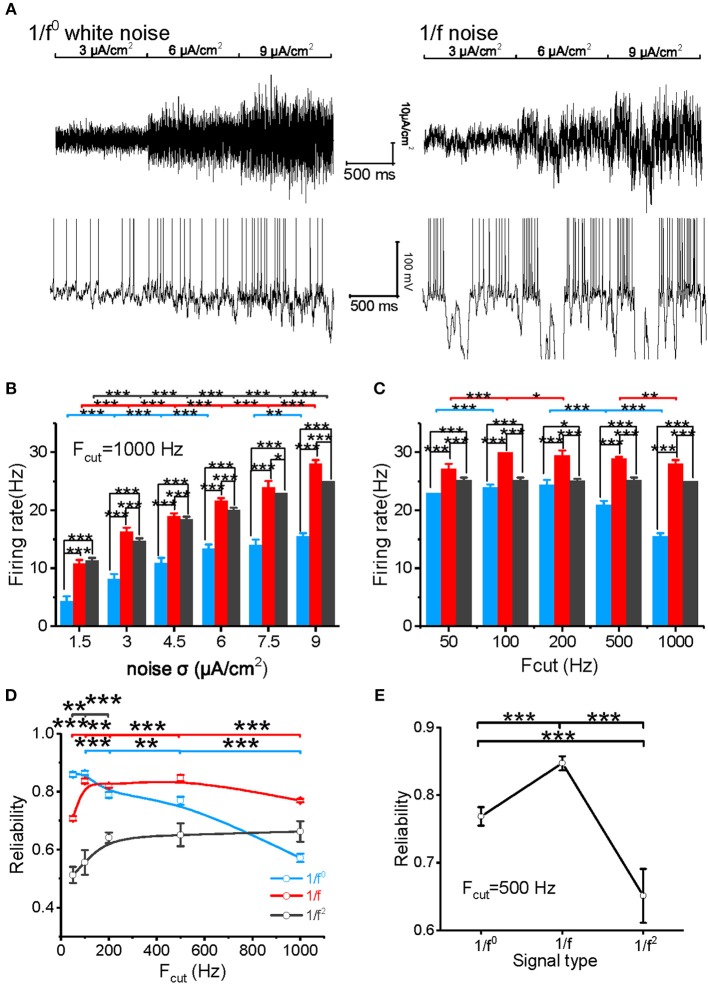
Model neuronal response to different types of input signal. **(A)** The top panel shows white noise (left) and 1/f noise (right) with step-changed variance introduced into the neuronal model. The bottom panel shows the membrane potentials of the model neuron in response to the input. The noise intensity shown here is 3, 6, and 9 μA/cm^2^. **(B)** Histogram of the neuronal firing rate vs. input signal SD for white noise (blue), 1/f noise (red), and 1/f^2^ noise (black) at the cutoff frequency of 1,000 Hz. **(C)** Histogram of the neuronal firing rate vs. cutoff frequencies (50, 100, 200, 500, and 1,000 Hz) for white noise (blue), 1/f (red), and 1/f^2^ noise (black) when the noise SD was 9 μA/cm^2^. **(D)** Plot of model neuronal reliability vs. cutoff frequencies (50, 100, 200, 500, and 1,000 Hz) for white noise (blue), 1/f (red), and 1/f^2^ noise (black). F_cut_, cutoff frequency. **(E)** Plot of reliability vs. β for the three types of 1/f^β^ input signals with a cutoff frequency of 500 Hz.

The model neuron first reproduced the similar responses to input signals with various noise intensities described in our experimental study (Figure 3A). For cutoff frequency of 1,000 Hz, the firing rates of neuronal model to white noise is significantly lower than that of 1/f and 1/f^2^ noise, and 1/f noise evokes the highest firing rate for the high intensities (Figures 3B,C). When the cutoff frequency increases from 50 to 1,000 Hz, the neuronal firing rate of model to input white noise decreases gradually. However, it increases slightly for 1/f noise, while it keeps almost invariant for 1/f^2^ noise (Figure 3C).

We also evaluated the spike reliability of the model neuron based on the evoked spike trains by repeatedly feeding the model neuron with each type of input signal with the different cutoff frequencies. Neuronal reliability for white noise is high only at a low cutoff frequency, while it decreases significantly with an increase in cutoff frequency (Figure 3D). On the contrary, neuronal response reliability to 1/f noise keeps at a much higher reliability level for almost the whole range of cutoff frequency (Figure 3D). In addition, the neuronal response reliability to 1/f^2^ noise is lower than the other two type of inputs (Figure 3E). However, its reliability increases as the cutoff frequency of 1/f^2^ noise increases (Figure 3D). These model simulation results are consistent with the experimental observations.

Next, we investigated the critical factors of neurons affecting the neuronal preference to 1/f signals. Considering that the responseness of neuronal model is dominated by the sodium and potassium channel kinetics, especially the channel opening velocity constant (e.g., α_m_, α_h_, α_n_) and channel closing velocity constant (e.g., β_m_ β_h_, and β_n_) (Yu et al., 2012), we systematically changed the values of these parameters to study their effects on neuronal preference to inputs. We have also varied the values of membrane input resistance R_input_ and the membrane capacitance C_m_ to understand the effect of the passive membrane property on the neuronal responseness. First we examined the effect of open (α_m_) and close (β_m_) velocity constants of sodium activation variable. Although neuronal firing rate could be significantly affected by an increase or decrease of the values of α_m_ and β_m_, the changing amount of the firing rate is almost same for all the three signal types (Supplementary Figures S3A,B).

**Figure 4:**
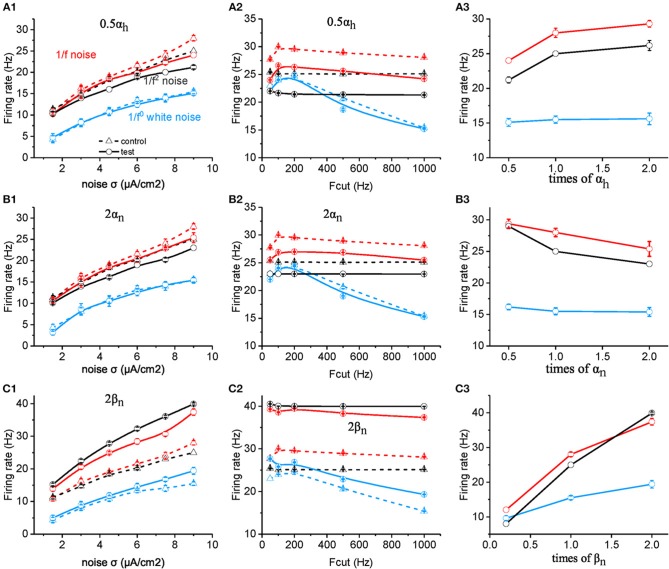
Effects of ionic channel kinetic constants on the responsive firing rate of model neurons. **(A1–C1)** Plots of firing rate vs. intensity for white noise (blue), 1/f (red), and 1/f^2^ noise (black) for a normal neuron (dotted line) compared with the results from the model neuron in the case of 0.5 α_h_ (solid line, **A1**), 2 α_n_ (solid line, **B1**), 2 β_n_ (solid line, **C1**). Fcut = 1,000 Hz. **(A2–C2)** Plots of firing rate vs. cutoff frequency for white noise (blue), 1/f (red), and 1/f^2^ noise (black) for a normal neuron (dotted line) compared with the results from the model neuron in the case of 0.5 α_h_ (solid line, **A2**), 2 α_n_ (solid line, **B2**), 2 β_n_ (solid line, **C2**). Noise σ = 9 μA/cm^2^. **(A3–C3)** Plots of firing rate vs. times of α_h_ (solid line, **A3**), α_n_ (solid line, **B3**), and β_n_ (solid line, **C3**) for white noise (blue), 1/f (red), and 1/f^2^ noise (black) for a normal neuron (dotted line) compared with the results from the model neuron. F_cut_ = 1,000 Hz. Noise σ = 9 μA/cm^2^.

However, when the value of α_h_ is halved, the neuronal firing rate as a function of noise intensity (Figure 4A1) or cutoff frequency (Figure 4A2) decreases dramatically for both 1/f and 1/f^2^ noise, but slightly for white noise. Figure 4A3 summarizes the contribution effect. When theα_h_ value increases, the neuronal firing rate increases gradually to a saturation level for both 1/f and 1/f^2^ noises while keeps invariant for white noise input. This suggests that α_h_ plays a role in the neuronal preference to 1/f signals. Next, we varied the value of β_h_ and observed almost no effect on neuronal firing rate (please see Supplementary Figure S3C).

Next, we examined the effect of α_n_ and β_n_ for the potassium channel activation variable. When the value of α_n_ was doubled (Figures 4B1,B2), the neuronal firing rate as a function of noise intensity (Figure 4B1) or cutoff frequency (Figure 4B2) decreased dramatically for both 1/f and 1/f^2^ noise, but slightly for white noise. Figure 4B3 summarizes the contribution effect, and shows that the firing rates of neuronal model decrease gradually when the α_n_ value increased for both 1/f and 1/f^2^ noises while deceased slightly for the white noise input. This suggests that α_n_ also contributes to the neuronal preference to 1/f signals.

Moreover, when β_n_ is increased, firing rate for 1/f^2^ noise increases greater than for 1/f noise, much more than for white noise, at each noise intensity (Figure 4C1) as well as for cutoff frequency (Figure 4C2). As a result, in the case of large β_n_, neuronal firing rate for 1/f^2^ noise is larger than 1/f noise, while in the case of small β_n_, neuronal firing rate for 1/f^2^ noise is lower than 1/f noise (Figure 4C3). This indicated that relatively large β_n_ value may dominate the neuronal preference to 1/f and 1/f^2^ noise. For the much small β_n_ value, neuronal preference to 1/f noise decreases dramatically.

**Figure 5:**
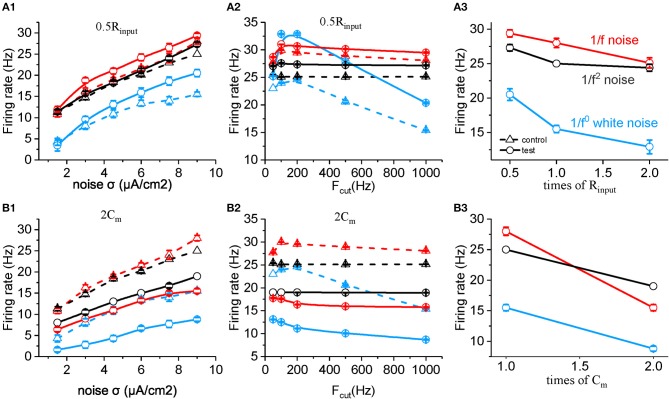
Effects of membrane time constants on the responsive firing rate of model neurons. **(A1,B1)** Plots of firing rate vs. intensity for white noise (blue), 1/f (red), and 1/f^2^ noise (black) for a normal neuron (dotted line) compared with the results from the model neuron in the case of 0.5 R_input_ (solid line, **A1**), 2 C_m_ (solid line, **B1**). Fcut = 1,000 Hz. **(A2,B2)** Plots of firing rate vs. cutoff frequency for white noise (blue), 1/f (red), and 1/f^2^ noise (black) for a normal neuron (dotted line) compared with the results from the model neuron in the case of 0.5 R_input_ (solid line, **A2**), 2 C_m_ (solid line, **B2**). Noise σ = 9 μA/cm^2^. **(A3,B3)** Plots of firing rate vs. times of R_input_ (solid line, **A3**), and C_m_ (solid line, **B3**) for white noise (blue), 1/f (red), and 1/f^2^ noise (black) for a normal neuron (dotted line) compared with the results from the model neuron. F_cut_ = 1000 Hz. Noise σ = 9 μA/cm^2^.

Based on the equation, the membrane time constant τ_c_ = R_input_^*^ C_m_, τ_c_ could be changed by either changing R_input_ or C_m_. Halved R_input_ increased neuronal firing rate to all the three type of input, and the neuronal sensitivity reached the maximum to white noise in the low cutoff frequency around 200 Hz, which is higher than to 1/f and 1/f^2^ noise stimuli (Figures 5A1,A2). Doubled C_m_ decreases the firing rate and makes the neuronal firing rate for 1/f^2^ noise larger than for 1/f noise at various noise intensity (Figure 5B1) and each cutoff frequency, respectively (Figure 5B2). As shown in Figures 5A,B, both C_m_ and R_input_ changed the neuronal firing rate for white noise more than for 1/f noise, while the change for 1/f^2^ noise is not that strong. This is contrary to the situation of changing β_n_. As β_nand_ R_input_ or C_m_ have different weighted effect on neuronal response, they may act together to support the neuronal preferential response to 1/f noise with a broad frequency range. So we concluded β_n_, R_input_ and C_m_ play important roles in neuronal responsive firing rate to 1/f^β^ noises.

**Figure 6:**
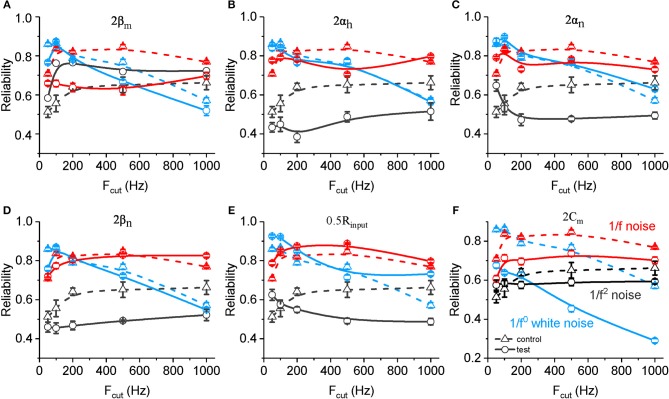
Effects of ionic and membrane time constants on the spike reliability of model neurons. **(A–F)** Plots of reliability vs. cutoff frequency for white noise (blue), 1/f (red) and 1/f^2^ noise (black) for a normal neuron (dotted line, **A–F**) compared with the results from the model neuron in the case of 2 β_m_ (solid line, **A**), 2 α_h_ (solid line, **B**), 2 α_n_ (solid line, **C**), 2 β_n_ (solid line, **D**), 0.5 R_input_ (solid line, **E**), and 2 C_m_ (solid line, **F**). F_cut_, cutoff frequency. R_input_, membrane input resistance. C_m_, membrane capacitance.

We also examined changes in the spike reliability in response to noises with altered neuronal biophysical parameters. As shown in Figure 6A, an increased value ofβ_m_ results in a significant decreased spike reliability for both 1/f noise (in all frequency range) and white noise (mainly with cutoff frequency higher than 200 Hz). On the contrary, increased β_m_ results in significantly increased spike reliability for 1/f^2^ noise. With doubled α_h_, α_n_, and β_n_ (Figures 6B–D) and decreased R_input_ (Figure 6E), neuronal spike reliability for 1/f^2^ noise decreases significantly for most of frequency range. These effects for 1/f^2^ noise are stronger than for 1/f noise, and much stronger for white noise. On the contrary, doubled C_m_ decreases the reliability significantly for each type of input, especially for the white noise (Figure 6F). Noted here, there is no clear change observed for the other rate constants (i.e., α_m_ and β_h_).

## Discussion

Recordings of neuronal membranes exhibit time-dependent voltage fluctuations (Destexhe et al., 2003; El Boustani et al., 2009), which have not been extensively studied in the last few decades due to the lack of a functional understanding of the underlying noise composition. In addition, 1/f^β^ noise has mainly been considered as background noise in previous studies (Mainen and Sejnowski, 1995; Nozaki and Yamamoto, 1998; Nozaki et al., 1999b; Jia et al., 2001; Ruszczynski et al., 2001; Richardson et al., 2003; Chizhov and Graham, 2008; Köndgen et al., 2008; Mino and Durand, 2008; Gutkin et al., 2009; Higgs and Spain, 2009; Sekine et al., 2009; Guo and Li, 2011; Sobie et al., 2011; Duan et al., 2014; Zhao et al., 2017). Here, for the first time, we directly examine cortical pyramidal neuronal responsiveness to 1/f^β^ (β = 0, 1, and 2) input signals. Our results shed light on the mechanism by which the statistical structure of input signals affects the dynamics of spike generation in cortical pyramidal cells.

### The Effect of the Frequency Range on the 1/f^β^ Noise Response

We compared neuronal responses to 1/f^0^, 1/f, and 1/f^2^ noises with various cutoff frequencies (50–1,000 Hz) and observed that cortical pyramidal cells exhibit a substantial loss of reliability and sensitivity to white noise when the cutoff frequency exceeds 200 Hz. Neuronal responsiveness to 1/f is also better than 1/f^2^ at various cutoff frequencies. In previous studies, white noise has been widely utilized to detect neuronal input and output functions (Sakai, 1992; Fairhall et al., 2001; Cook et al., 2007; Vilela and Lindner, 2009). Based on our results, 1/f noise should be a much better probe for determining the response properties of neurons at various input frequency ranges, instead of white noise or 1/f^2^ noise, and the signal cutoff frequency should be carefully established in future studies.

Our work suggests that the neuronal firing rate and reliability for 1/f noise are both enhanced with an increase in the cutoff frequency. Thus, each frequency interval has an effect on neuronal responses, and future work should examine the optimum signal frequency range for neuronal responsiveness and derive the neuronal input-output function for each frequency interval. Although a previous study has found that injecting white noise to the DC component increases neuronal spike reliability (Mainen and Sejnowski, 1995), our findings with zero mean signals imply that neuronal excitability may determine the effects of signal frequency range, and ultimately modulate neuronal response.

### Preferential Neuronal Response to 1/f Noise With a Large Frequency Range

The 1/f noise is a common phenomenon in nature (Bak et al., 1987; Gilden et al., 1995; Musha and Yamamoto, 1997; Novikov et al., 1997; De Coensel et al., 2003; Bédard et al., 2006; He, 2014). In the brain, EEG and ECoG recordings reveal that when neuronal populations exhibit the 1/f characteristic, the neural network is capable of highly efficient information processing (Soma et al., 2003; Lin and Chen, 2005; Shew and Plenz, 2013; Wood et al., 2016). At the cellular level, 1/f noise was found to originate from intact network inputs (El Boustani et al., 2009), and can enhance neuronal excitability and the stochastic resonanGarciace effect (Nozaki and Yamamoto, 1998; Nozaki et al., 1999a,b; El Boustani et al., 2009). Based on neuronal sensitivity to low-frequency sine wave signals (Hutcheon et al., 1996; Hunter et al., 1998; Volgushev et al., 1998; Fellous et al., 2001; Yu et al., 2001; Brumberg, 2002; Richardson et al., 2003; Köndgen et al., 2008; Levi et al., 2015), an individual neuron uses signals with a low frequency range to process information. However, recent experiments have observed high frequency components in the recorded membrane potentials of cortical neurons *in vivo* (El Boustani et al., 2009; Bedard et al., 2017).

Our results, for the first time, show that neurons respond preferentially to the 1/f noise in large frequency ranges, with respect to the firing rate and spike timing reliability. Because the rate and temporal encoding are two major encoding methods for neurons (Nowak et al., 1997; Reinagel and Reid, 2000; Fellous et al., 2001; Brette and Guigon, 2003; Avissar et al., 2007; Freund and Cerquera, 2012) and neuronal activities at all levels show 1/f characteristics *in vivo* (Bédard et al., 2006; El Boustani et al., 2009; Freeman and Zhai, 2009; Milstein et al., 2009; Dehghani et al., 2010; He et al., 2010; Ciuciu et al., 2012; Pettersen et al., 2014; Voytek et al., 2015), our results may indicate the positive effects of the 1/f property and large frequency range on the neuronal responses for normal physiological functioning. In addition, since the 1/f type of signal substantially drives neuronal response, our findings suggest new operating rules for synaptic transmission, neuronal plasticity and other activities relying on neuronal firing. It is likely that future studies of neuronal interactions and network activities will discover additional distinct effects of 1/f probe signals on neural information process and neural computation.

### Neuronal Dynamics Underlying the 1/f Preference

Given the low-pass filter property of neuronal membrane, once the signal contain more power in low frequency range, it may induce more neuronal firing, as seen from the neuronal response to 1/f compared with to white noise (Fellous et al., 2001; Brumberg, 2002; Levi et al., 2015). However, this is in contradiction with our finding that 1/f noise evokes higher firing rate than 1/f^2^ noise in a large frequency rage. Especially, we observed that neuronal firing rate for 1/f noise increases with the cutoff frequency of 1/f noise increases. In addition, it was reported previously that the refractory period endows neuron with high-pass filtering (Nozaki et al., 1999a), increasing the response complexity. We performed numerical simulations by using the Hodgkin-Huxley model to gain deep insights into the mechanism underlying the neuronal responsive preference for the 1/f noise. We found that β_n_, R_input_, and C_m_ determine the neuronal preference to input signal with different type of statistics. Figure 4 shows that the effect of changing β_n_ on the neuronal firing rate to 1/f^2^ is larger to 1/f, much larger than to 1/f^0^ noise. On the contrary, the effects of changing R_input_ or C_m_ on the neuronal firing rate is stronger to 1/f^0^ noise than 1/f, even stronger than to 1/f^2^ inputs. Here, close rate constant of potassium activation β_n_ seems to form a high-pass filter effect, while both R_input_ and C_m_ act as low-pass filter effect. Their appropriately combined action may result in neuronal responsive preference for 1/f noise with a broad frequency range.

In addition, we found the neuronal responsive reliability for 1/f^β^ signal types is majorly dominated by several key factors of ion channels and membrane time constants, respectively. Specifically, as shown in results section, the spike reliability for white noise is mainly determined by C_m_. Spike reliability for 1/f and 1/f^2^ noise is mainly dominated by sodium activation close rate constant (α_m_), inactivation open rate constant (α_h_) and both open (α_n_), and close (β_n_) rate constants of potassium channel, as well as membrane time constant. With doubled α_h_, α_n_, and β_n_ (Figures 6B–D) and decreased R_input_ (Figure 6E), neuronal spike reliability for 1/f^2^ noise decreases significantly for most of frequency range. These effects for 1/f^2^ noise are stronger than for 1/f noise, and much stronger for white noise. These results revealed that the membrane capacitance dominates the neuronal preference to low frequency component, while the combined ion channel kinetics dominates the neuronal preference to high frequency components in the 1/f^β^ type noise stimuli.

A prevailing hypothesis in neuronal response is that the stochastic opening and closing of individual ion channels endows cortical neurons an inherent noise. When the correlation time of the external input matches the time scale of the inherent noise, the neuronal responsiveness is maximized. Indeed, previous studies have demonstrated the existence of the optimum time scale of input signals for neuronal spiking reliability (Galán et al., 2008; McGinley et al., 2015). The neuron-preferred 1/f signal in our finding is consistent with this hypothesis.

In addition, cortical neurons are also enriched in other subtypes of sodium, potassium, and calcium channels, which may play important roles in precisely controlling the cellular sensitivity to the input signals with different temporal correlations (Wang et al., 2003). Additionally, a balance between excitatory and inhibitory synaptic inputs may adjust neuronal excitability to different signal statistics (Brunel et al., 2001; Chance et al., 2002; Wang, 2010). The dendritic morphology of the cell may even enhance the neuronal sensitivity to some frequency components within input signals (Eyal et al., 2014; Ostojic et al., 2015). Future studies should examine how the synaptic balance and the interaction between the neuronal intrinsic dynamics and modulations from the recurrent network contribute to the neuronal responses to input signals with various higher order statics and cutoff frequencies.

In summary, as the signal cutoff frequency progressively increases from 50 to 1,000 Hz, the neuronal responsive firing rate and reliability increase for 1/f noise, but decrease for white noise. Ion channel kinetic and membrane time constants endow neurons with a preferential response for 1/f noise with high cutoff frequencies. These results suggest that the 1/f noise is important in determining the computational rules and operating principles of cortical circuits.

## Author Contributions

YY supervised the research. YY, GQ, and BF designed the research. GQ and XF performed the experimental research. GQ and YY wrote the paper. All authors performed data analysis and reviewed the manuscript.

### Conflict of Interest Statement

The authors declare that the research was conducted in the absence of any commercial or financial relationships that could be construed as a potential conflict of interest.
