# Supplementary material for: The Impact of Frequency Scale on the Response Sensitivity and Reliability of Cortical Neurons to 1/fβ Input Signals
Source: Front Cell Neurosci. 2019 Jul 11;13:311. doi: 10.3389/fncel.2019.00311 (PMC6637762; doi:10.3389/fncel.2019.00311)
Supplement: Supplementary file 1 [file Data_Sheet_1.PDF]

Supplementary figure S1. Neuronal responsive firing rate for white noise (blue), 1/f (red) and $1/f^2$ noise (black) with various intensities (82.37, 164.80, 247.23, 329.67, 412.10 and 494.53 pA) and a noisy cutoff frequency of 50 Hz (A), 100 Hz (B), 200 Hz (C) and 500 Hz (D). F_{cut}, cutoff frequency.

Supplementary figure S2. Plots of reliability vs β for the three types of $1/f^{\beta}$ input noises with a cutoff frequency of 50 Hz (A), 200 Hz (B), and 1000 Hz (C). F_{cut}, cutoff frequency.

Supplementary figure S3. Effects of α_m, β_m and β_h on the responsive firing rate of model neurons. (A1-C1) Plots of firing rate vs intensity for white noise (blue), 1/f (red) and $1/f^2$ noise (black) for a normal neuron (dotted line) compared with the results from the model neuron in the case of 2 α_m (solid line, A1), 2 β_m (solid line, B1), 5 β_h (solid line, C1). Fcut = 1000 Hz. (A2-C2) Plots of firing rate vs cutoff frequency for white noise (blue), 1/f (red) and $1/f^2$ noise (black) for a normal neuron (dotted line) compared with the results from the model neuron in the case of 2 α_m (solid line, A2), 2 β_m (solid line, B2), 5 β_h (solid line, C2). Noise σ = 9 $\mu A/cm^2$. (A3-C3) Plots of firing rate vs times of α_m (solid line, A3), β_m (solid line, B3) and β_h (solid line, C3) for white noise (blue), 1/f (red) and $1/f^2$ noise (black) for a normal neuron (dotted line) compared with the results from the model neuron. F_{cut} = 1000 Hz. Noise σ = 9 $\mu A/cm^2$.

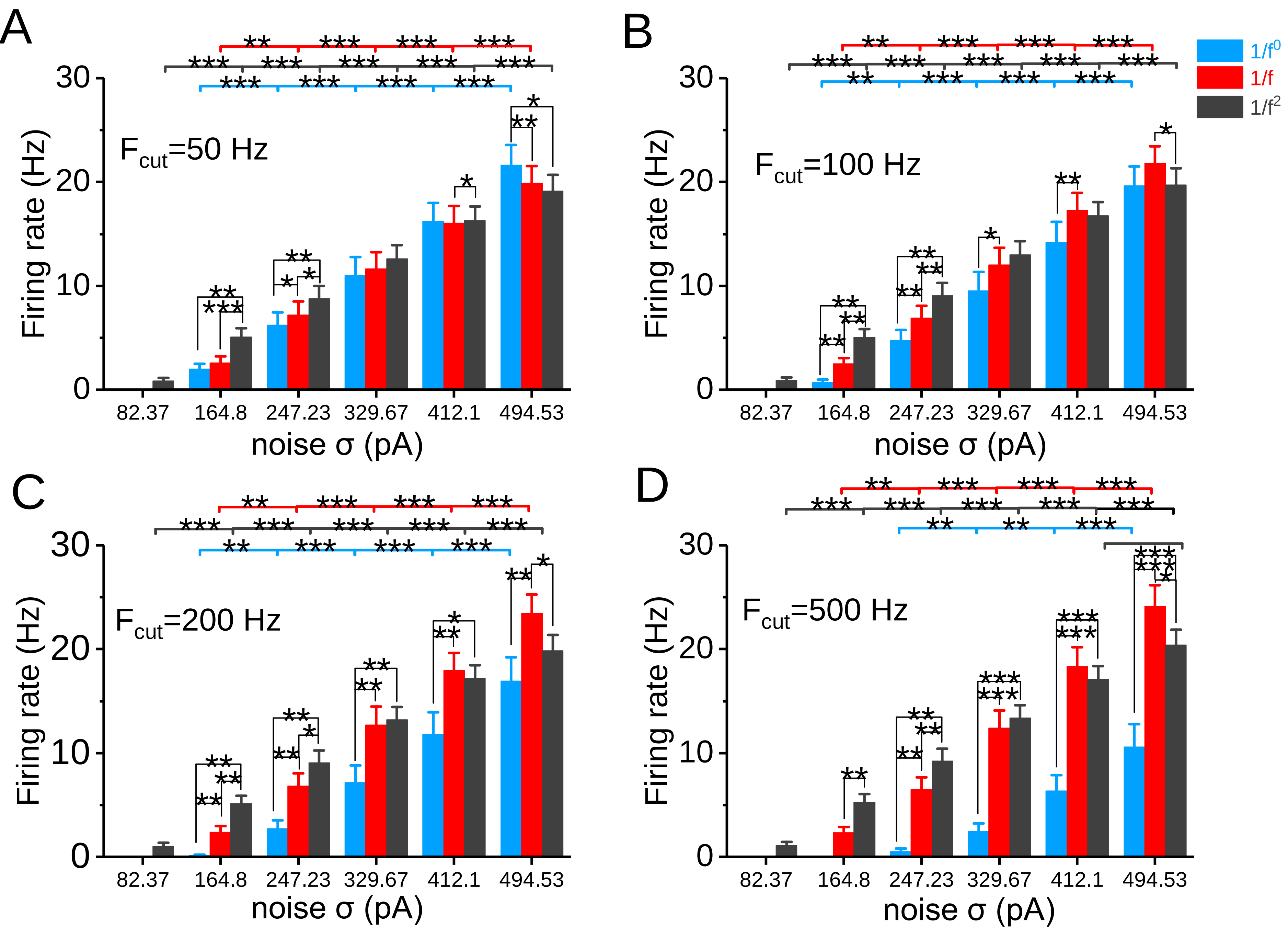

A
B
C
D
1/f0
1/f
1/f2
Fcut=50 Hz
Fcut=100 Hz
Fcut=200 Hz
Fcut=500 Hz
Firing rate (Hz)
noise σ (pA)
0
10
20
30
82.37
164.8
247.23
329.67
412.1
494.53

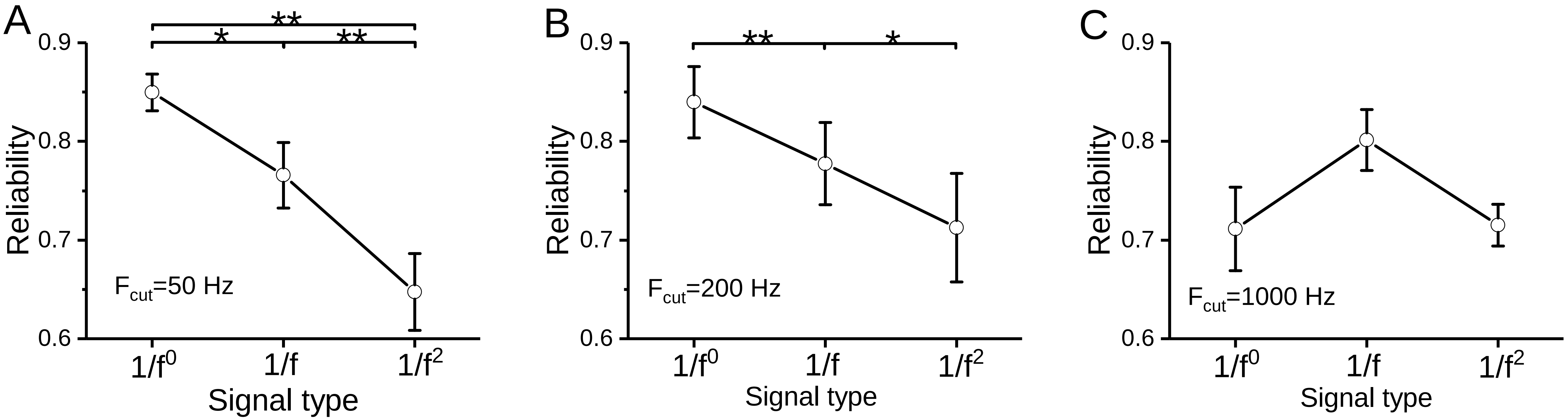
A
B
C
**
*
**
**
*
0.9
0.8
0.7
0.6
Reliability
F_cut=50 Hz
F_cut=200 Hz
F_cut=1000 Hz
1/f^0
1/f
1/f^2
Signal type

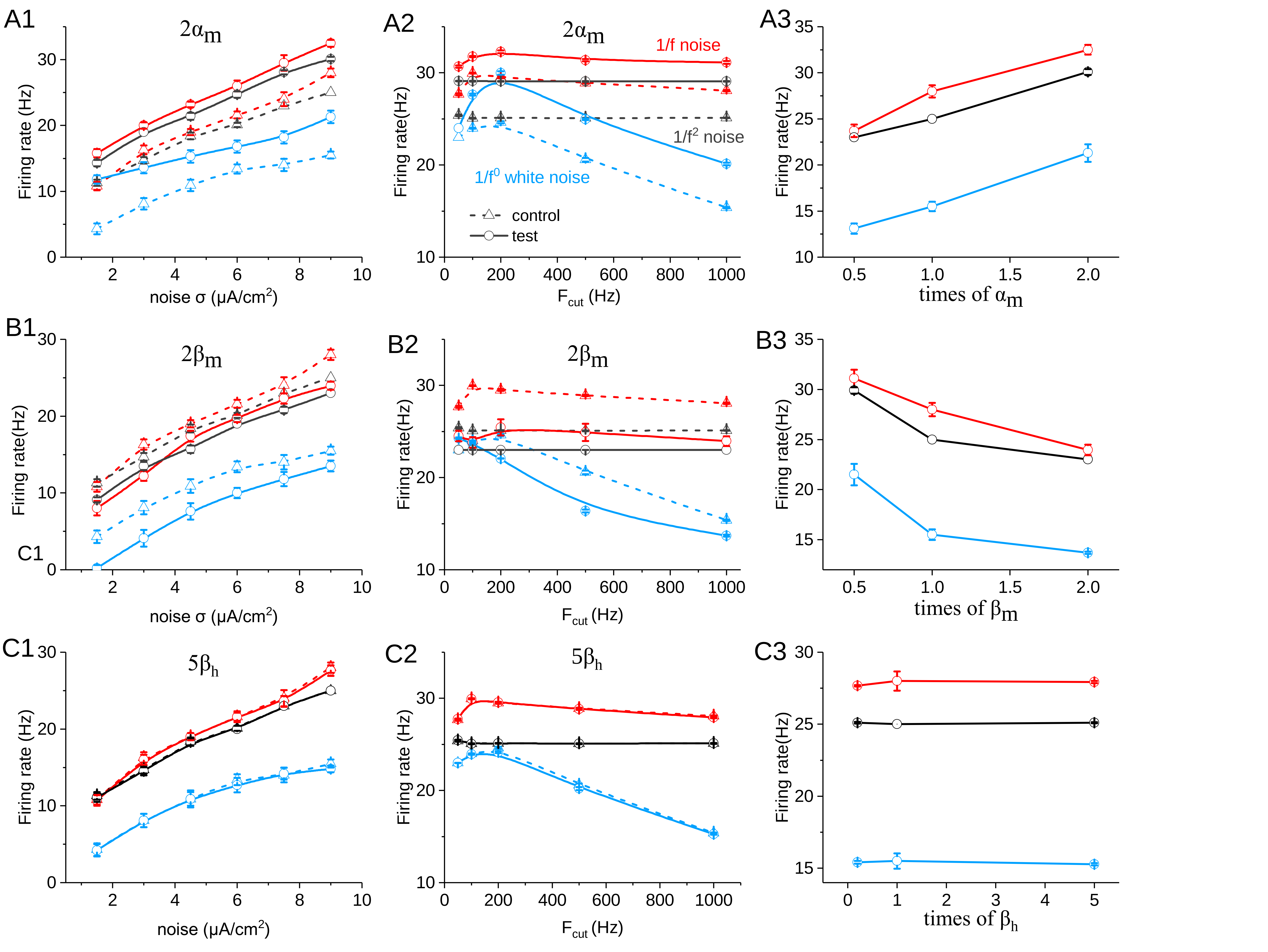

A1
$2\alpha_m$
Firing rate (Hz)
noise σ (μA/cm^2)
A2
$2\alpha_m$
1/f noise
1/f^2 noise
1/f^0 white noise
control
test
Firing rate(Hz)
F_{cut} (Hz)
A3
Firing rate(Hz)
times of α_m
B1
$2\beta_m$
Firing rate(Hz)
noise σ (μA/cm^2)
B2
$2\beta_m$
Firing rate(Hz)
F_{cut} (Hz)
B3
Firing rate(Hz)
times of β_m
C1
$5\beta_h$
Firing rate (Hz)
noise (μA/cm^2)
C2
$5\beta_h$
Firing rate (Hz)
F_{cut} (Hz)
C3
Firing rate(Hz)
times of β_h
